# Molecular Mechanism of Mouse Uterine Smooth Muscle Regulation on Embryo Implantation

**DOI:** 10.3390/ijms232012494

**Published:** 2022-10-18

**Authors:** Rui Cao, Zhen-Shan Yang, Sui-Li Hu, Shi-Jin Liang, Shu-Miao Zhang, Song-Qi Zhu, Lin Lu, Cheng-Hong Long, Si-Tong Yao, Yong-Jiang Ma, Xiao-Huan Liang

**Affiliations:** College of Veterinary Medicine, South China Agricultural University, Guangzhou 510642, China

**Keywords:** implantation, uterus, SMCs, actin, Tagln

## Abstract

Myometrium plays critical roles in multiple processes such as embryo spacing through peristalsis during mouse implantation, indicating vital roles of smooth muscle in the successful establishment and quality of implantation. Actin, a key element of cytoskeleton structure, plays an important role in the movement and contraction of smooth muscle cells (SMCs). However, the function of peri-implantation uterine smooth muscle and the regulation mechanism of muscle tension are still unclear. This study focused on the molecular mechanism of actin assembly regulation on implantation in smooth muscle. Phalloidin is a highly selective bicyclic peptide used for staining actin filaments (also known as F-actin). Phalloidin staining showed that F-actin gradually weakened in the CD-1 mouse myometrium from day 1 to day 4 of early pregnancy. More than 3 mice were studied for each group. Jasplakinolide (Jasp) used to inhibit F-actin depolymerization promotes F-actin polymerization in SMCs during implantation window and consequently compromises embryo implantation quality. Transcriptome analysis following Jasp treatment in mouse uterine SMCs reveals significant molecular changes associated with actin assembly. Tagln is involved in the regulation of the cell cytoskeleton and promotes the polymerization of G-actin to F-actin. Our results show that Tagln expression is gradually reduced in mouse uterine myometrium from day 1 to 4 of pregnancy. Furthermore, progesterone inhibits the expression of Tagln through the progesterone receptor. Using siRNA to knock down Tagln in day 3 SMCs, we found that phalloidin staining is decreased, which confirms the critical role of Tagln in F-actin polymerization. In conclusion, our data suggested that decreases in actin assembly in uterine smooth muscle during early pregnancy is critical to optimal embryo implantation. Tagln, a key molecule involved in actin assembly, regulates embryo implantation by controlling F-actin aggregation before implantation, suggesting moderate uterine contractility is conducive to embryo implantation. This study provides new insights into how the mouse uterus increases its flexibility to accommodate implanting embryos in the early stage of pregnancy.

## 1. Introduction

Embryo implantation, a critical step in mammalian reproduction, is the process in which an activated blastocyst and a receptive uterus interact and establish a close connection. It involves an intricate series of molecular and cellular interactions, all of which must be accomplished within an optimal implantation time [1]. Early pregnancy failure in humans is usually due to defects occurring in the peri-implantation. Despite the rapidly growing population, which may reach 9 billion by 2050, 15% of couples worldwide are still childless due to infertility, which is a global social and economic problem [1]. Therefore, the research in embryo implantation is of great significance.

The mouse uterus mainly consists of uterine epithelia, stroma, and myometrium [2]. Among them, myometrium, which is classified as smooth muscle, is an important component. Smooth muscle cells (SMCs), as the main cells in smooth muscle, ensure the contractility of the mammalian uterus [3]. SMCs are highly plastic and participate in regulating cell contraction, proliferation, and extracellular matrix synthesis [4]. Actin is one of the key elements of cytoskeletal structure in SMCs and plays a crucial role in cell function and motility [5]. It can exist in both monomeric globular actin (G-actin) and polymeric filamentous actin (F-actin) [6]. G-actin rapid polymerizes into F-actin to promote cell contraction [7], which plays crucial roles in SMC motility [8,9]. Proper regulation of cytoskeletal dynamics is critical for initiating dialogue between embryo and maternal endometrium during implantation [10]. Consistent in multiparous mammals, embryos implant in an evenly distributed manner along the uterine longitudinal axis [11,12,13,14,15]. From a developmental and physiological perspective, it prevents embryo overcrowding and embryo loss due to unnecessary nutritional and spatial competition. Previous studies have shown that embryo transport may be mediated by uterine mechanical force, which is generated by myometrium movement. Therefore, peristalsis mediated by uterine contractions is critical for embryo spacing along the uterus [16].

Transgelin (also known as smooth muscle protein 22α, SM22α/Tagln) is an abundant smooth muscle-specific 22 kD protein that acts as an early marker of smooth muscle tissue [17]. It is localized in the cytoskeleton and expressed in endothelial cells, SMCs, fibroblasts, and various immune cells [18]. Tagln can bind to actin as a regulator of cytoskeletal structures. In addition, Tagln participates in cytoskeleton remodeling by facilitating the polymerization of G-actin protein into F-actin, which has long been hypothesized to have the function of regulating cell development and contraction [19]. The main determinants of cell contraction are actin and actin-binding proteins. Actin requires actin-binding proteins such as Tagln to regulate actin assembly to function [20]. Tagln is necessary to maintain uterine contractility during mice parturition, and knocking out Tagln leads to reduced F-actin stress fibers and impaired mitochondrial morphology and function, resulting in dystocia. In addition, mouse uterine SMCs undergo phenotypic modulation during pregnancy, similar to the phenotypic regulation of vascular SMCs [21]. However, the role of Tagln in early pregnancy is still not clear. This study aims to contribute a more nuanced understanding of myometrium regulation on embryo implantation.

## 2. Results

### 2.1. Changes in Actin Assembly in the Mice Uterus during Early Pregnancy

**Figure 1:**
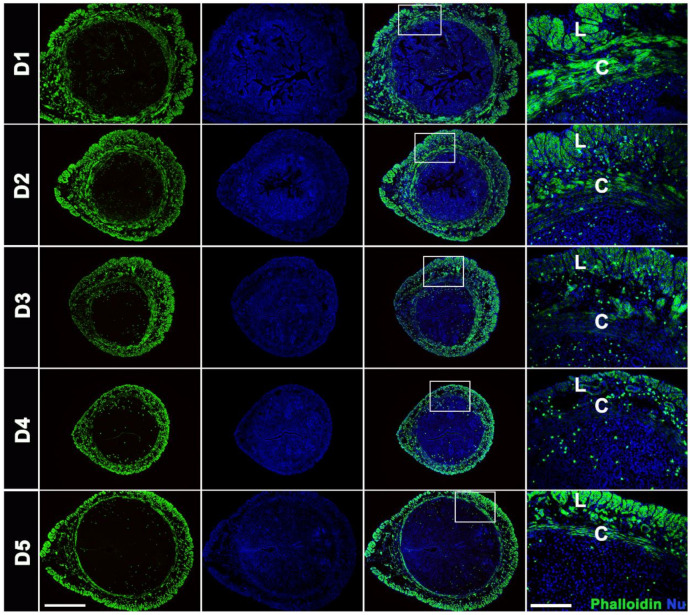
Phalloidin staining in mice uterus of early pregnancy. Phalloidin staining in mice myometrium on days 1 to 5 of pregnancy, Phalloidin (green), the nuclei were counterstained using DAPI (blue). L, longitudinal muscle layer; C, circular muscle layer. Scale bars = 200 µm, 500 µm.

To explore changes in actin assembly in mice, we examined the expression of F-actin by phalloidin staining. The results showed that phalloidin staining signals in the myometrium were gradually weakened during days 1 to 4 of early pregnancy (Figure 1 and Figure S1). On day 5 of pregnancy, levels of F-actin in circular muscle began to recover (Figure 1).

### 2.2. Effects of Actin Assembly in the Smooth Muscle on Mouse Embryo Implantation

**Figure 2:**
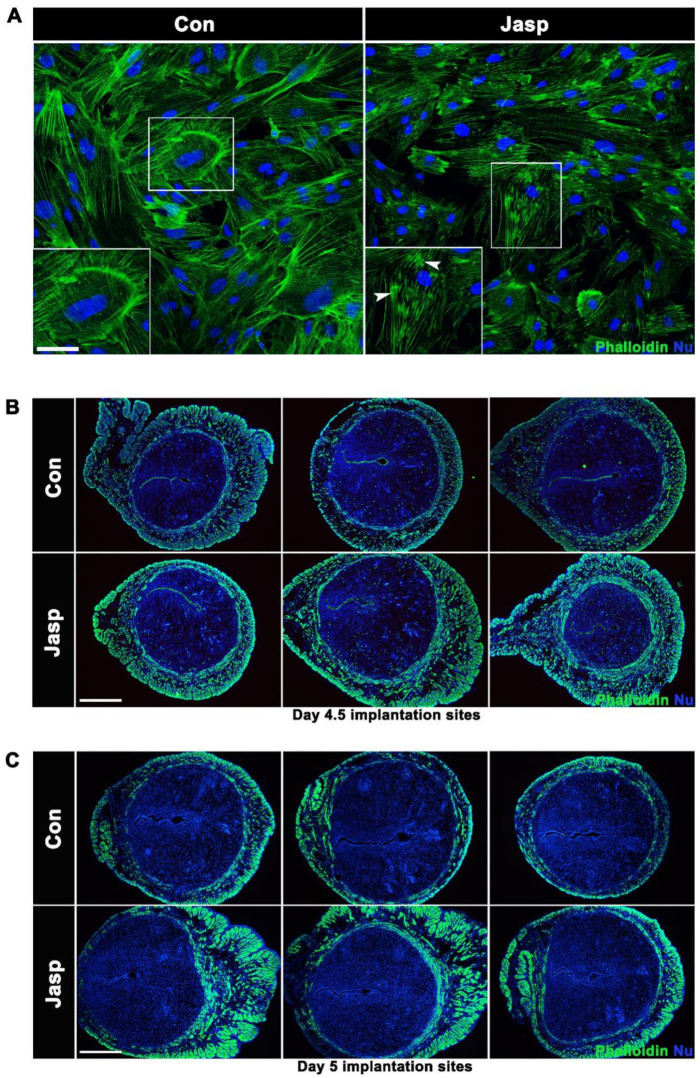
Jasp promotes F-actin accumulation in uterine smooth muscle. (**A**) Phalloidin staining of mice uterine SMCs treated with 100 nM Jasplakinolide for 24 h on day 3 of pregnancy. Scale bars = 200 µm. (**B**,**C**) Mice were intraperitoneally injected with Jasp 10 µg/mice at 09:00 am on day 3 of pregnancy, and uteri were collected on days 4.5 and 5 of pregnancy. Phalloidin (green), DAPI (blue). Scale bars = 500 μm.

Jasp, a F-actin depolymerization inhibitor [22,23], mouse uterine SMCs on day 3 of pregnancy were treated with 100 nM Jasp to increase smooth muscle tension. The results showed that aggregated F-actin bundles were increased in SMCs treated with Jasp for 24 h (Figure 2A).

**Figure 3:**
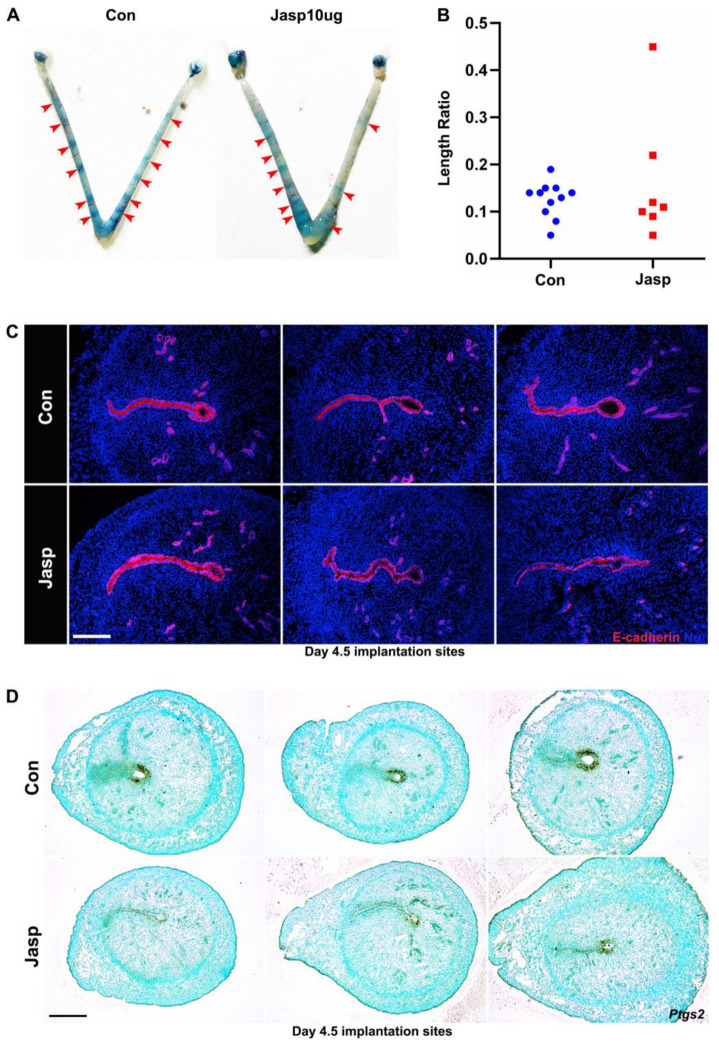
Abnormal embryo implantation on day 4.5 of pregnancy after Jasp treatment. (**A**) The mice were intraperitoneally injected with Jasp 10 µg/mice on day 3 of pregnancy, and embryo distribution was observed on day 4.5 of pregnancy (red arrows). (**B**) The ratio of embryo interval distance to full uterine length on day 4.5 of pregnancy. More than 6 mice were studied for each group. (**C**) Abnormal embryo morphology on day 4.5 of pregnancy, E-cadherin (red) labeled epithelial cells, DAPI (blue), with three replicates in the figure. Scale bars = 200 µm. (**D**) In situ hybridization results for *Ptgs2* on day 4.5 of pregnancy, with three replicates in the figure. Scale bars = 500 µm.

To study the regulatory effect of uterine smooth muscle tension on embryo implantation, mice were injected intraperitoneally with Jasp 10 µg/mice at 9:00 am on day 3 of pregnancy. Phalloidin staining in the Jasp-treated groups was stronger than the control groups in the uteri on days 4.5 and 5 of pregnancy (Figure 2B,C), proving that Jasp stabilizes F-actin bundles. We next examined embryo implantation on day 4.5 of pregnancy. Results showed that embryos were unevenly distributed in the Jasp-treated group compared with the control group (Figure 3A). Inter-implantation uterine lengths were quantified and normalized against the full uterine length. The distance between implantation sites in the Jasp-treated group showed a wider variance (Figure 3B). Immunofluorescence results of the epithelial cell marker E-cadherin showed irregular shapes of embryo implantation sites in the Jasp group. In comparison with symmetrically oval-shaped implantation sites, Jasp implantation sites were smaller with tortuous boundaries and mesometrial-antimesometrial axis on day 4.5 of pregnancy (Figure 3C). Prostaglandin-endoperoxide synthase 2 (Ptgs2) is a marker of embryo attachment reaction. In situ hybridization showed that *Ptgs2* expression was downregulated in Jasp group (Figure 3D).

**Figure 4:**
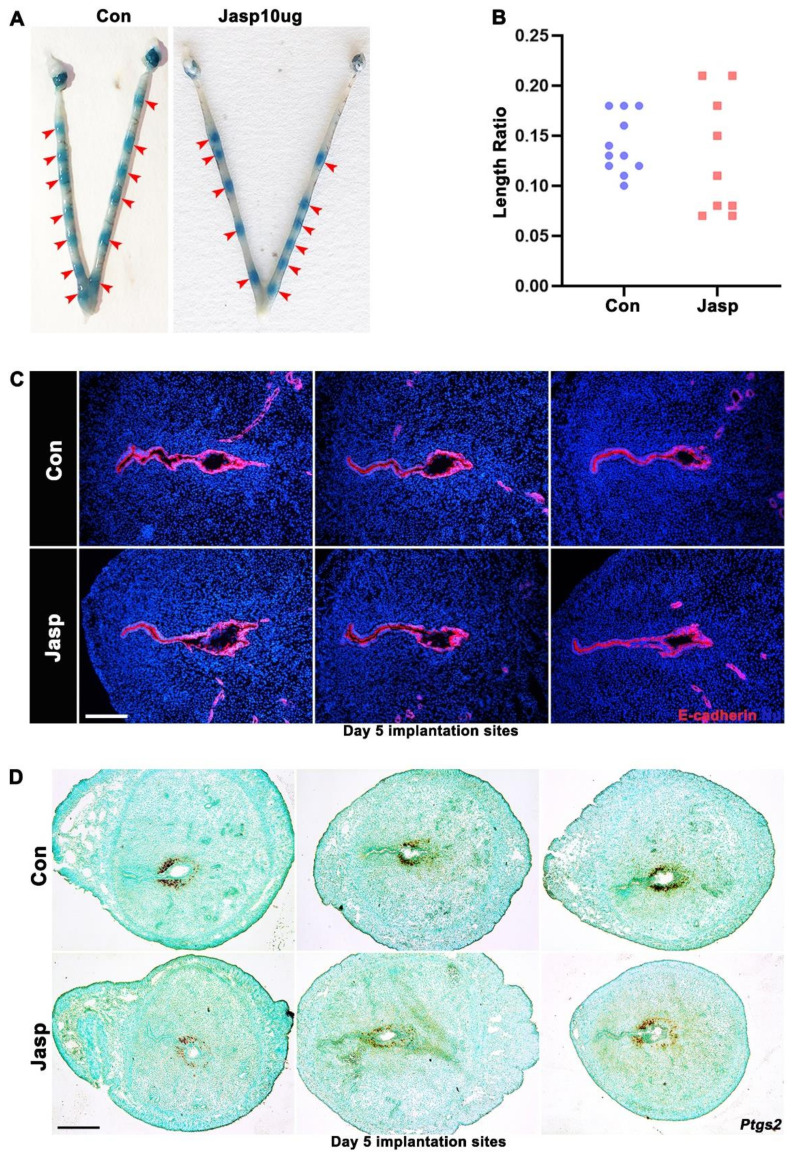
Abnormal embryo implantation on day 5 of pregnancy after Jasp treatment. (**A**) The mice were intraperitoneally injected with Jasp 10 µg/mice on day 3 of pregnancy, and embryo distribution was observed on day 5 of pregnancy (red arrows). (**B**) The ratio of embryo interval distance to full uterine length on day 5 of pregnancy. More than 6 mice were studied for each group. (**C**) Abnormal embryo morphology on day 5 of pregnancy, E-cadherin (red), DAPI (blue), with three replicates in the figure. Scale bars = 200 µm. (**D**) In situ hybridization results for *Ptgs2* on day 5 of pregnancy, with three replicates in the figure. Scale bars = 500 μm.

These observations were further confirmed by the examination of embryo implantation on day 5 of pregnancy after Jasp treatment. The results showed that embryo spacing was unevenly distributed in the Jasp group (Figure 4A), and the ratio of embryo interval distance to full uterine length in the Jasp group was significantly different compared with the control group (Figure 4B). Previous studies have shown that shaped crypt cavity formation is probably key to successful implantation [24,25]. The typical spearhead shape in control implantation chambers was disrupted in Jasp group (Figure 4C). In situ hybridization results showed that *Ptgs2* expression was attenuated on day 5 of pregnancy in the Jasp-treated uteri (Figure 4D), which was consistent with the expression of *Ptgs2* on day 4.5 of pregnancy. Combined with the above results, it was concluded that the data suggests that an abnormally elevated contractile function in uterine smooth muscle adversely affects embryo implantation.

### 2.3. Differentially Expressed Genes in Uterine Smooth Muscle after Jasp Treatment

**Figure 5:**
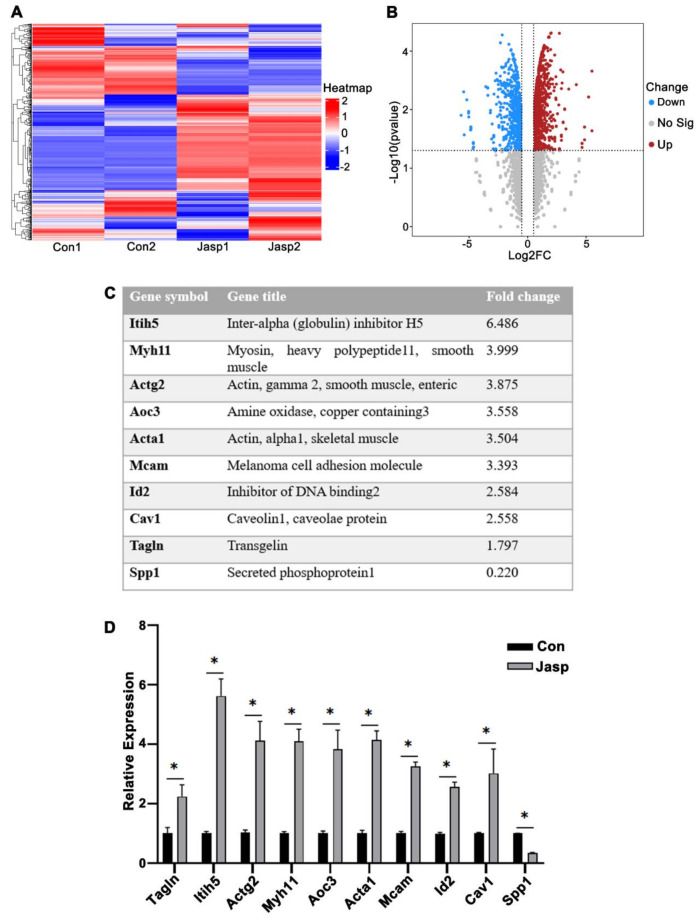
Differentially expressed genes after Jasp treatment of SMCs. (**A**) RNA−seq analysis of mice uterine SMCs on day 3 of pregnancy treated with 100 nM Jasp for 24 h. (**B**) The volcano map showed differentially expressed genes in con and Jasp groups, upregulated genes (red dot), and down-regulated genes (blue dot). (**C**) Molecules associated with actin assembly in sequencing results. (**D**) Verify the mRNA expression level of the changing molecule. * *p* < 0.05.

To investigate molecular mechanisms regulating actin assembly in smooth muscle, transcriptome was analyzed using mouse uterine SMCs on day 3 of pregnancy after being treated with Jasp for 24 h. Heat map analysis showed that gene expression levels were significantly different between Jasp group and the control group (Figure 5A). As shown in the volcano plot, 744 genes were up-regulated, and 641 genes were down-regulated (Figure 5B).

Analysis of the sequencing results revealed that a group of molecules related to actin assembly were upregulated in Jasp-treated SMCs including inter-alpha (globulin) inhibitor H5 (Itih5), actin gamma 2 (Actg2), amine oxidase, copper containing 3 (Aoc3), actin alpha 1 (Acta1), melanoma cell adhesion molecule (Mcam), DNA binding inhibitor 2 (Id2), caveolin 1 (Cav1), and myosin heavy polypeptide 11 (Myh11), while secreted phosphoprotein 1 (Spp1) was reduced (Figure 5C). RT-qPCR results were consistent with the sequencing analysis results (Figure 5D). These results revealed potential molecule targets to further study the mechanistic regulation of uterine muscle contraction in early pregnancy.

### 2.4. Expression of Tagln in Early Pregnant Mouse Uterus

**Figure 6:**
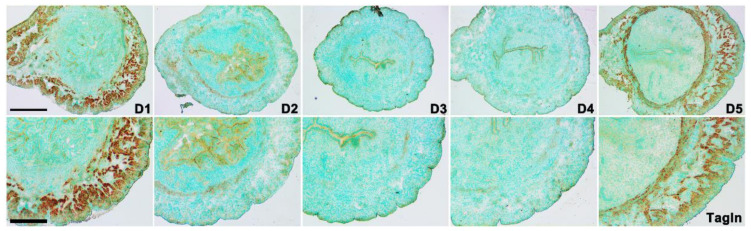
Expression of *Tagln* in mice uterus from days 1 to 5 of early pregnancy. In situ hybridization to detect the expression of Tagln in mice uterus on days 1 to 5 of pregnancy. Scale bars = 200 μm, 500 μm.

Tagln is involved in cytoskeleton regulation, promoting G-actin polymerization to F-actin, so we focused on the expression of Tagln in mouse early pregnancy. In situ hybridization results showed that *Tagln* was expressed in uterine smooth muscle on days 1 to 5 of pregnancy, and expression levels declined from day 1 to day 4 of pregnancy. On day 5, the expression of Tagln enhanced remarkably (Figure 6). In summary, we found that the expression pattern of Tagln is associated with the pattern of F-actin in early pregnancy. Therefore, it is speculated that Tagln regulates uterine smooth muscle tension by mediating actin assembly, thereby participating in embryo implantation process.

### 2.5. Steroid Hormone Regulation on Tagln

**Figure 7:**
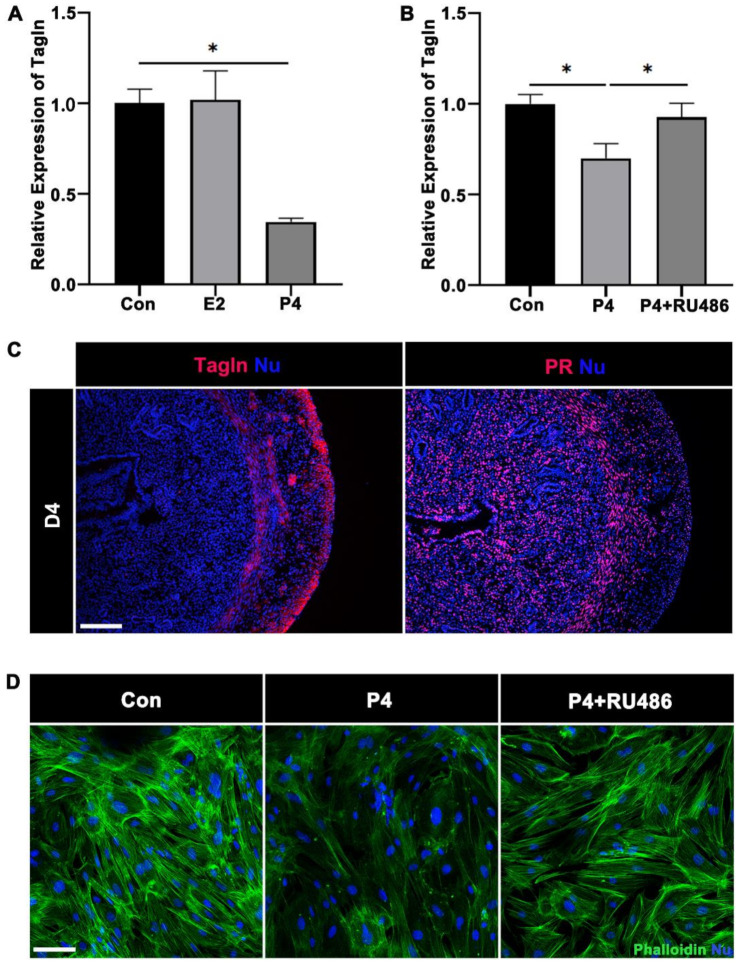
P4 regulates the expression of Tagln through PR. (**A**,**B**) The mRNA expression level of Tagln was measured at 20 nM E2, 100 nM P4, and 1 μM RU486 + P4 for 48 h of uterine SMCs on day 4 of pregnancy, * *p* < 0.05. (**C**) Immunofluorescence of Tagln (red) and PR (red) with continuous mice uterine slices on day 4 of pregnancy. Scale bars = 200 μm. (**D**) Phalloidin staining (green) of mouse uterine SMCs on day 4 of pregnancy treated with 100 nM P4, 1 μM RU486 + P4 for 48 h. Scale bars = 200 μm.

To detect whether Tagln is a downstream molecule regulated by steroid hormones, mouse uterine SMCs on day 4 of pregnancy were treated with E2 and P4 for 48 h. RT-qPCR results showed that the mRNA expression of *Tagln* decreased 48 h after P4 treatment, while *Tagln* expression in the E2 treatment group did not change significantly (Figure 7A), suggesting that Tagln is regulated by progesterone. SMCs were treated with P4 plus progesterone receptor (PR) antagonist RU486 for 48 h. Results showed that the inhibitory effect of progesterone on *Tagln* was abolished (Figure 7B). Furthermore, immunofluorescence was performed on continuous uterine sections on day 4 of pregnancy. The results showed that PR expression was stronger in circular muscle than longitudinal muscle. In contrast, Tagln expression in circular muscle was weaker than that in longitudinal muscle (Figure 7C). Phalloidin staining results showed that weakened actin bundles were decreased in the P4 treatment group, and were enhanced in P4 plus RU486 group, which was consistent with the RT-qPCR results (Figure 7D). In conclusion, P4 inhibits the expression of Tagln through PR, thereby regulating actin assembly in SMCs.

### 2.6. Tagln Regulates the Assembly State of F-Actin in Smooth Muscle

**Figure 8:**
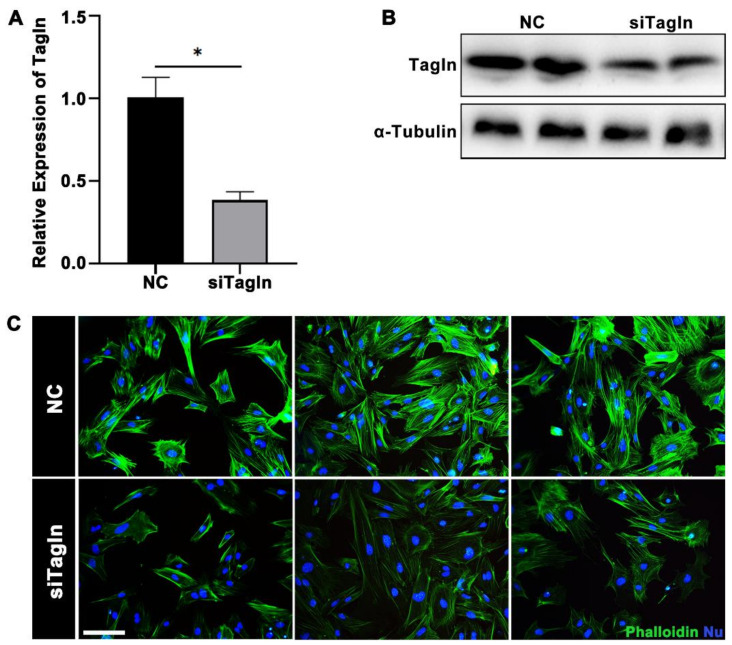
Reduced F-actin polymerization after transfected siTagln in SMCs. (**A**,**B**) RT-qPCR and western blot were used to detect the interference efficiency of Tagln in mice uterine SMCs on day 3 of pregnancy, * *p*  <  0.05. (**C**) Phalloidin staining (green) after siTagln transfection, triplicate results. Scale bars = 200 μm.

To explore roles of Tagln on actin assembly in uterine smooth muscle, we knockdown *Tagln* by transfection with siRNAs. RT-qPCR and western blotting results showed that *Tagln* siRNA significantly downregulates the expression of *Tagln* in SMCs (Figure 8A,B). Phalloidin staining showed that inhibition of *Tagln* caused reduced staining signal intensity (Figure 8C). The results showed that Tagln plays a role in embryo implantation by regulating F-actin polymerization and uterine smooth muscle tension.in early pregnancy in mouse.

## 3. Discussion

Uterine movement is mediated by myometrium, and normal myometrium movement is essential for embryo implantation and continued pregnancy [16,26]. Previous studies have found that myometrium contractile movements help establish appropriate conditions for embryo implantation [26,27]. Myometrium peristaltic contractions play a vital role in embryo spacing. Abnormal uterine peristalsis can lead to embryo implantation with abnormal spacing, which can lead to miscarriage or other pregnancy complications such as placenta previa, which is directly related to reduced pregnancy rates in humans [28,29]. It has been shown that LPA3 regulates embryo spacing by affecting uterine contractions, and LPA3-specific agonists can induce rapid uterine contractions in wild-type mice [30]. Moreover, some studies have found that uterine peristalsis is markedly decreased during embryo implantation, which is believed to help embryo implantation into endometrium [31,32,33].

Actin, a key element of cytoskeleton structure, plays an important role in the movement and contraction of SMCs. G-actin and F-actin are two forms of actin. In SMCs, actin is in dynamic equilibrium and is involved in regulating SMCs contraction. The transition from G-actin to F-actin is responsible for providing cell contractility and motility [8]. Our study found that phalloidin staining in mouse myometrium gradually decreased during days 1 to 4 of pregnancy. On day 5, phalloidin staining was recovered. Day 4 of pregnancy in mice serves as a key node for embryo implantation. At this stage, the uterus is in a receptive state, conducive to embryo implantation. The decrease in phalloidin staining during this period suggests that declined F-actin polymerization is accompanied by weakened smooth muscle tension during the implantation window.

Jasp acts as an F-actin stabilizer that stabilizes F-actin turnover and inhibits the G-actin pool in SMCs [34]. In addition, Jasp induces F-actin network redistribution and can induce apoptosis in various cells, including immune cells, fibroblasts, and cancer cells [35]. To clearly understand the role of actin assembly in early pregnancy, phalloidin staining of mouse uterine SMCs after Jasp treatment revealed that many aggregated F-actin appeared in the cytoskeleton, indicating that the actin polymerizer Jasp can facilitate F-actin polymerization. The results showed that phalloidin staining was enhanced in Jasp group mice uterus. Compared with the control group, embryo distribution became uneven in the Jasp group. The implantation crypts and embryo morphology were aberrant and lost normal spearhead shape in the Jasp-treated group. Detecting implantation-related molecule *Ptgs2* found that *Ptgs2* expression was notably reduced on days 4.5 and 5 of pregnancy. These results indicate that abnormal actin assembly in uterine smooth muscle promotes excessive myometrial contraction, resulting in uneven embryo distribution, and thus affecting the quality of embryo implantation. Therefore, we propose that moderate uterine contractility is conducive to embryo implantation.

To understand the potential molecular mechanism of actin assembly, RNA samples of SMCs treated with Jasp were sequenced, and the RNA-seq results were analyzed to find some significant differences in molecular expression related to actin assembly regulation. Mcam and Aoc3 belong to myocardia-related transcription factors (MRTFs) and serum response factors (SRFs) [36]. MRTFs are strong activators of SRF-dependent target genes involved in contraction, movement, and adhesion [37]. Moreover, the products of canonical MRTFs, including smooth muscle marker molecules Myh11, Actg2, Acta1, and Tagln, are closely related to actin assembly and play a role in regulating the contractile movement of SMCs. Cytoplasmic Cav-1 could form a pivotal structural component for caveolae. The actin cytoskeleton and caveolae are important players in regulating cell morphology and directional migration [38]. Early studies revealed the association of caveolae with F-actin [39,40], and the actin-binding protein filamin A was found to be important for caveolae co-alignment with actin stress fibers [41]. The role of Id2 is mainly manifested in promoting cell cycle progression to control cell proliferation in SMCs [42]. Spp1, also known as osteopontin, which is a down-regulated molecule, can be secreted by smooth muscle. Spp1 is a highly upregulated extracellular matrix/adhesion molecule/cytokine in the human uterine receptive phases. Compared with wild-type mice, Spp1 knockout mice exhibited reduced pregnancy rates in the second trimester [43]. Furthermore, some studies have shown that Spp1 has the potential to affect tissue remodeling at the fetal-maternal interface by influencing cell-cell and cell-extracellular matrix dialogue, increasing cell proliferation, migration, and regulating local cytokine pathways [44].

Tagln acts as an actin cytoskeleton modulator and promotes F-actin polymerization. The expression of *Tagln* was examined by in situ hybridization. *Tagln* expression in mice myometrium gradually decreased in myometrium on days 1 to 4 of pregnancy. The decreasing trend of Tagln in myometrium was more significant in circular muscle layer than in longitudinal muscle. This pattern was similar to phalloidin staining pattern in mouse uteri from day 1 to 5 of pregnancy. Taken together, we showed that Tagln plays an important role in embryo implantation as an actin assemble regulator, but the molecular mechanism of Tagln in embryo implantation is currently unclear.

In rodents, smooth muscle activity is well regulated by E2 and P4. Studies have found that steroid hormones and glucocorticoids regulate gene expression and proliferation in SMCs [45,46,47,48]. P4 through nuclear PR regulates various genes expression during uterine implantation. At present, it is clear that P4 plays a complex role in myometrial during pregnancy. For example, in 1956, Csapo proposed a vital role of P4 in pregnancy was to “block” myometrium contraction, and thus needed to withdraw this blocker when labor begins [45]. High-frequency uterine contractions adversely affect pregnancy and implantation rates in in vitro fertilization-embryo transfer, so the effect of hormones on uterine contractility was also investigated in the human non-pregnant uterus and in IVF cycles prior to embryo transfer [31]. Furthermore, P4 signaling is absolutely necessary for the uterus to obtain tolerance for embryo implantation and pregnancy maintenance [49]. To analyze whether steroid hormones regulate Tagln, mouse uterine SMCs were treated with E2 and P4 on day 4 of pregnancy, respectively, and we found that *Tagln* expression was declined in the P4-treated group, but not notably changed in the E2-treated group. To determine whether Tagln was regulated by P4, SMCs were treated with P4 plus RU486. The results showed that RU486 alleviated the inhibitory effect of P4 on *Tagln*. In mouse uteri on day 4, immunofluorescence results showed that PR was mainly expressed in circular muscle, while Tagln was reduced in circular muscle. Moreover, phalloidin staining was reduced in the P4-treated group, which promotes the idea that the downregulation of Tagln under P4 inhibition would reduce F-actin polymerization, thereby receding SMCs contractility.

Many studies found that uterine muscle contraction in response to oxytocin was significantly reduced in *Tagln* knockout mice during pregnancy [50]. In addition, severe mitochondrial damages, such as mitochondrial swelling, cristae destruction, and even disappearance, were observed in uterine smooth muscle during term pregnancy in *Tagln* knockout mice, which eventually led to mitochondrial membrane potential collapse and impaired ATP synthesis [50]. These studies suggest that Tagln is necessary to maintain uterine contractility during parturition in mice. At the same time, some studies found that Tagln-deficient mice are fertile and develop normally, but with reduced smooth muscle contractility and significant changes in actin filament distribution and cytoskeleton organization [51,52]. In another study, phalloidin staining intensity was reduced after transfected TAGLN-shRNA in human bone marrow-derived stromal stem cells [53], illustrating the inextricable link between Tagln and F-actin. Our cellular fluorescence results showed that phalloidin staining intensity was attenuated after transfected siRNA against Tagln in SMCs, confirming that Tagln plays an important role in regulating actin assembly, that promoting F-actin polymerization. This study sheds light on a previously unrecognized role of Tagln in mouse implantation, which promotes F-actin polymerization in uterine smooth muscle, thus regulating smooth muscle tension and influencing embryo implantation. *Tagln* conditional knockout in mouse uterine smooth muscle may provide more evidence for its function during implantation and subsequent pregnancy outcomes, but the lack of conditional Tagln knockout mice prevents further verification. As a molecule of Tagln family, Tagln2 has been shown to function in embryo implantation by promoting actin polymerization [10]. Therefore, we speculate that Tagln and Tagln2 are involved in embryo implantation and regulate uterine smooth muscle throughout pregnancy and parturition. However, we have not yet investigated the effect of Tagln on decidualization and later stages, and these ideas may be further explored in future studies.

Taken together, we reveal the molecular mechanism by which actin assembly in mouse uterine smooth muscle during early pregnancy is involved in regulating embryo implantation. F-actin decreases in the uterine smooth muscle on day 4 of pregnancy, it is possible that the myometrium contraction during implantation is moderate to distribute embryo evenly. Elevated F-actin polymerization increases the contraction of uterine smooth muscle, which is unfavorable for implantation, thus affecting the quality of embryo implantation. However, more potential molecular mechanisms regulating actin assembly remain to be explored.

## 4. Materials and Methods

### 4.1. Animals and Treatments

Mature CD-1 mice were housed in an SPF animal room with 12 h of light and 12 h of darkness. All animal procedures were approved by the Institutional Animal Care and Use Committee of South China Agricultural University (No. 2021f108). Female mice, aged 6–8 weeks, were mated with fertile males to induce pregnancy (day 1 is the day of vaginal plug). On days 1–4, pregnancy was confirmed by flushing embryos from oviducts or uterus. On days 4.5 and 5, the implantation sites were visualized through intravenous injection of 0.1 mL of 1% Chicago blue dye (Sigma Aldrich, St. Louis, MO, USA) in saline.

### 4.2. Jasplakinolide Injection

To determine the role of actin in embryo implantation. Female mice were intraperitoneally injected with Jasplakinolide (Jasp, 10 μg/mouse in 0.1 mL saline, Tocris) on day 3 of pregnancy (09:00 am). The control mice were injected intraperitoneally with saline (0.1 mL/mouse). Then, pregnant mice were sacrificed on day 4.5 (1:00–2:00 am) and day 5 (9:00 am) to collect the uterine tissues. The implantation sites of activated uteri were identified through intravenous injection of Chicago blue dye.

### 4.3. In Situ Hybridization

Total RNAs from the mouse bladder were reverse transcribed and amplified with the specific primers for mouse *Tagln*. pGEMT plasmid (Promega) was used for cloning the amplified fragments of *Tagln* and verified by sequencing. The primers of T7 and SP6 were used to amplify *Tagln* fragment from the pGEM-Tagln plasmids. Digoxigenin-labeled antisense or sense cRNA probes were transcribed in vitro using a digoxigenin RNA labeling kit (Roche Applied Science, Penzberg, Germany). In situ hybridization was performed as previously described [54]. In brief, frozen uterine sections (10 μm) were mounted on (3-Aminopropyl) triethoxysilane (A3648, Sigma Aldrich, St. Louis, MO, USA) treated slides and fixed in 4% paraformaldehyde solution in phosphate-buffered saline (PBS). Hybridization was performed at 55℃ for 16 h. Digoxigenin-labeled *Tagln* sense probe was used as a negative control. Following hybridization and post-hybridization washes, sections were incubated with sheep anti-digoxigenin antibody conjugated to alkaline phosphatase (1:5000, Roche Applied Science, Penzberg, Germany). Endogenous alkaline phosphatase activity was inhibited with 2 mM levamisole (Sigma-Aldrich, St. Louis, MO, USA). Sections were stained with 1% methyl green for 3 min. The positive signal was visualized as the dark brown color.

### 4.4. Isolation and Culture of Mice Uterine Smooth Muscle Cells

Uterine horns from days 3 and 4 in pregnant mice were split longitudinally, washed with Hanks’ balanced salt solution (HBSS, Sigma, St. Louis, MO, USA), and digested with 5% trypsin (Amresco, Cleveland, OH, USA) and 6 mg/mL dispase (Roche Applied Science, Penzberg, Germany) in HBSS for 1 h at 4 °C followed by 1 h at room temperature and 10 min at 37 °C. After washing three times with HBSS, the remaining uteri were incubated in 6 mL HBSS containing 0.15 mg/mL collagenase I (Invitrogen, Waltham, MA, USA; 17100-017) at 37 °C for 35 min. The uteri were shaken by hand and washed three times with HBSS, then treated with 3 mL 0.5% trypsin and 0.5% collagenase for 1 h at 37 °C. The digested uteri were shaken, and charcoal-treated fetal bovine serum (cFBS, Biological Industries, Cromwell, CT, USA) terminated the digestion. The uteri were then filtered using 70 μm wire filter gauze. Filtrated was centrifuged at 1200 rpm for 10 min. After discarding supernatant, cells were washed with HBSS and then resuspended in 5 mL DMEM/F12 (Sigma-Aldrich, St. Louis, MO, USA) medium containing 10% cFBS, cultured at 37 °C for 4 h, and then replaced with DMEM/12 containing 10% cFBS to remove other non-adherent cells.

### 4.5. siRNA Transfection

The siRNA for mouse Tagln and nonspecific siRNA were designed and synthesized by Ribobio Co., Ltd. (Guangzhou, China). The mice uterine SMCs on day 3 of pregnancy were transfected with siRNA using Lipofectamine 3000 (Invitrogen, Waltham, MA, USA) according to the manufacturer’s protocol, and cells were transfected for 24 h. These siRNA sequences were listed in Table 1.

### 4.6. Jasplakinolide Treatments

Mouse uterine SMCs on day 3 of pregnancy were treated with 100 nM jasplakinolide, and the control group was added with dimethyl sulfoxide. After culturing at 37 °C for 24 h, cells were collected for RNA extraction or fixed for phalloidin staining according to experimental needs.

### 4.7. Analysis of Differentially Expressed Genes

The RNAs differential expression analysis was performed by DESeq2 software between two different groups [55]. The genes/transcripts with the parameter of false discovery rate <0.05 and absolute fold change ≥2 were considered differentially expressed genes/transcripts.

### 4.8. Hormone Treatments

Mice uterine SMCs on day 4 of pregnancy were starved with serum-free DMEM/F12 for 12 h, then were treated with 20 nM 17β-estradiol (E2, Sigma-Aldrich, St. Louis, MO, USA) and 100 nM progesterone (P4, Sigma-Aldrich, St. Louis, MO, USA) in serum-free DMEM/F12 for 24 h. Progesterone receptor antagonist RU486 (1 mM, Sigma-Aldrich, St. Louis, MO, USA, M8046) and P4 were treated together for 48 h.

### 4.9. Real-Time Quantitative Polymerase Chain Reaction (RT-qPCR)

Total RNAs from mouse uterine SMCs were isolated using TRIzol reagent (TaKaRa), RNA was reversed transcribed into cDNA with HiScript II Reverse Transcriptase kit (Vazyme), and then RT-qPCR was performed using ChamQTM Universal SYBR^®^ qPCR Master Mix (Vazyme) on the CFX96 Touch Real-Time PCR Detection system (BioRad). All reactions were run in triplicate. Table 1 shows the primer sequences. The specificity of PCR product amplification was assessed by analysis of the melting curve. Gene expression was normalized with housekeeping genes, ribosomal protein L7 (Rpl7). As both genes were stable, the ΔΔCT method was employed to determine relative changes in gene expression compared to Rpl7.

### 4.10. Immunofluorescence

Frozen sections (10 μm) heat-fixed at 55 °C for 3 min. Then fix with 4% paraformaldehyde for 10 min. It was then washed three times in PBST (0.01% Triton X-100) for 10 min each, blocked with blocking solution at 37 °C for 30 min, and then with goat anti-Tagln antibody (1:200, Abcam, Cambridge, UK, ab10135), rat anti-E-cadherin antibody (1:500, Abcam, Cambridge, UK, ab11512), rabbit anti-PR antibody (1:400, CST, 8757 s) overnight at 4 °C. The results were observed directly after staining with phalloidin (1:1000, Sigma-Aldrich, St. Louis, MO, USA, P5282). The next day, it was washed 3 times with PBST and incubate with anti-rabbit, anti-goat, anti-rat IgG conjugated to Alexa Fluor 594 (Jackson ImmunoResearch, William Seagrove, PA, USA) for 30 min at room temperature, then washed 3 times with PBST. Nuclei were counterstained with 6-diamino-2-phenylindole (DAPI, Sigma-Aldrich, St. Louis, MO, USA). The results were observed under a fluorescence microscope (Leica).

### 4.11. Western Blot

Western blot was performed as previously described [19]. Briefly, cells were lysed in lysis buffer RIPA (Beyotime biotechnology). Protein concentration was quantified using the BCA kit (Sigma-Aldrich, St. Louis, MO, USA). Samples were then resolved on a 10% SDS-PAGE gel and transferred onto PVDF membranes (Millipore). After blocking with 5% skim milk (Sigma-Aldrich, St. Louis, MO, USA), membranes were incubated with primary antibody for Tagln (1:5000, Abcam, Cambridge, UK) overnight at 4 °C Next, secondary antibody conjugated with HRP (1:5000, Invitrogen, Waltham, MA, USA) was added to membranes for 1 h at room temperature. Signals were detected with the ECL kit (Pierce).

### 4.12. Statistical Analysis

Statistical analyses were performed using the Student’s t-test when analysis the differences between the two groups. Analysis of variance (ANOVA) was performed for multiple comparisons. A value of *p* < 0.05 was considered statistically significant. The data were presented as the mean ± standard deviation. And all the experiments were independently repeated at least three times.

## Figures and Tables

**Table 1:** Primer sequences were used in this study.

Gene	Sequence (5′-3′)	Product Size	Application	Accession Number
Tagln	TCTCCTTCCAGTCCACAA	233 bp	ISH	NM_011526.5
	TGCTCAGAATCACACCATT			
Tagln	GTGTGATTCTGAGCAAATTGGTG	202 bp	RT-qPCR	NM_011526.5
	ACTGCTGCCATATCCTTACCTT			
Itih5	ATCAACTGCATGTGGAGGTCA	103 bp	RT-qPCR	NM_172471.2
	ACTGAGACATCATTCCCCATCT			
Myh11	ATGAGGTGGTCGTGGAGTTG	170 bp	RT-qPCR	NM_001161775.2
	GCCTGAGAAGTATCGCTCCC			
Actg2	CCGCCCTAGACATCAGGGT TCTTCTGGTGCTACTCGAAGC	192 bp	RT-qPCR	NM_009610.2
Aoc3	GAAGACCACCCTAGTGCTCCT ATGAAGAGGTTGGCTCAGTCC	112 bp	RT-qPCR	NM_009675.2
Acta1	TACCACCGGCATCGTGTTG GCGCACAATCTCACGTTCAG	190 bp	RT-qPCR	NM_001272041.1
Acta2	GGCACCACTGAACCCTAAGG ACAATACCAGTTGTACGTCCAGA	135 bp	RT-qPCR	NM_007392.3
Mcam	CCTTCCTGCCTGCTACTA CTGTCCTGTCTCTTGTTACTA	201 bp	RT-qPCR	NM_001359530.1
Id2	ATGAAAGCCTTCAGTCCGGTG AGCAGACTCATCGGGTCGT	107 bp	RT-qPCR	NM_010496.3
Tagln	GCTGAAGAATGGTGTGATT		siRNA	

## Data Availability

All the data generated in this study are included in this manuscript.

## Supplementary Materials

The supporting information can be downloaded at: https://www.mdpi.com/article/10.3390/ijms232012494/s1.

## Abbreviations

Actin gamma 2	Actg2
Amine oxidase, copper containing 3	Aoc3
Actin alpha 1	Acta1
Caveolin 1	Cav1
DNA binding inhibitor 2	Id2
Filamentous actin	F-actin
Globular actin	G-actin
Inter-alpha (globulin) inhibitor H5	Itih5
Melanoma cell adhesion molecule	Mcam
Myosin heavy polypeptide 11	Myh11
Myocardia-related transcription factors	MRTFs
Progesterone receptor antagonist	RU486
Prostaglandin-endoperoxide synthase 2	Ptgs2
Ribosomal protein L7	Rpl7
Smooth muscle cells	SMCs
Secreted phosphoprotein 1	Spp1
Serum response factors	SRFs
Transgelin	Tagln
