# Supplementary material for: Molecular Mechanism of Mouse Uterine Smooth Muscle Regulation on Embryo Implantation
Source: Int J Mol Sci. 2022 Oct 18;23(20):12494. doi: 10.3390/ijms232012494 (PMC9604262; doi:10.3390/ijms232012494)
Supplement: Supplementary file 1 [file ijms-23-12494-s001.zip › Figure S1.pdf]

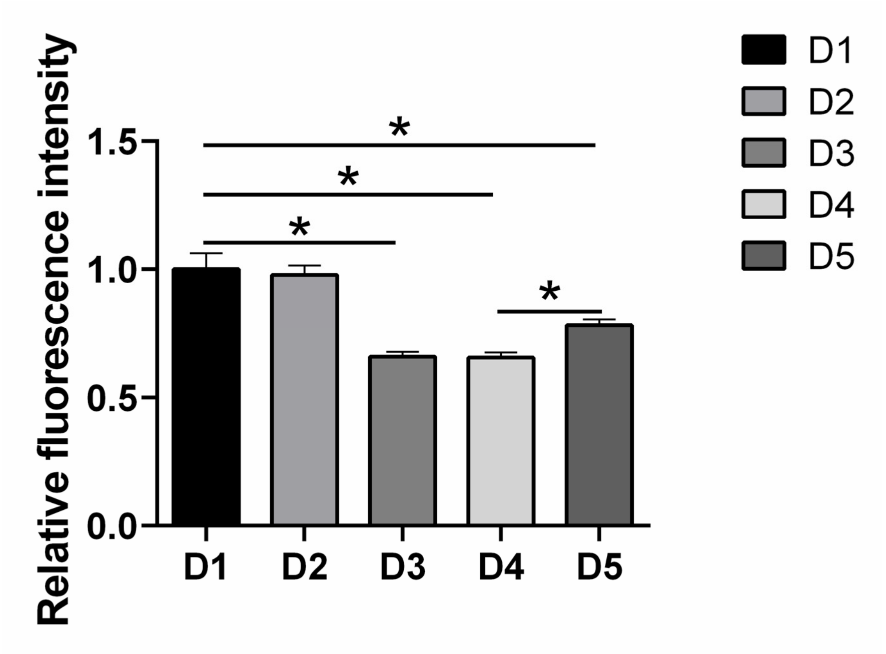

Figure S1. Quantification of the phalloidin staining in mouse uteri during the early pregnancy. Bars represent mean ± SD (*p-value < 0.05).
